# *Parachlamydia* and *Rhabdochlamydia* in Premature Neonates

**DOI:** 10.3201/eid1512.090267

**Published:** 2009-12

**Authors:** Frédéric Lamoth, Sébastien Aeby, Antoine Schneider, Katia Jaton-Ogay, Bernard Vaudaux, Gilbert Greub

**Affiliations:** University Hospital and University of Lausanne, Lausanne, Switzerland

**Keywords:** Parachlamydia, Rhabdochlamydia, PCR, bacteria, amebae, respiratory secretions, neonates, letter

**To the Editor:** New members have recently been recognized in the order Chlamydiales (*1*). The family *Rhabdochlamydiaceae* includes *R. porcellionis* (a parasite of *Porcellio scaber*) and *R. crassificans* (a pathogen of the cockroach *Blatta orientalis*) (*2*,*3*); their pathogenic role in humans has not yet been investigated. *Parachlamydia acanthamoebae* and *Protochlamydia naegleriophila* belong to the family *Parachlamydiaceae* (*1*,*4*). Increasing evidence indicates that these obligate intracellular bacteria infecting free-living amebae may cause respiratory diseases in humans (*1*). Recent findings also suggest a role for *Parachlamydia* in miscarriage, stillbirth, and preterm labor (*5*–*7*). Whether these bacteria may contaminate the newborns of infected mothers is unknown.

The aims of this study were to 1) develop a real-time PCR for detecting *Rhabdochlamydia* spp. and 2) apply this PCR, and those previously described for *Parachlamydia* and *Protochlamydia* (*4*,*8*), to respiratory samples from premature neonates. Using the GenBank database (www.ncbi.nlm.nih.gov), we selected primers RcF (5′-GACGCTGCGTGAGTGATGA-3′) and RcR (5′-CCGGTGCTTCTTTACGCAGTA-3′), and probe RcS (5′-6 carboxyfluorescein-CTTTCGGGTTGTAAAACTCTTTCGCGCA-Black Hole Quencher 1-3′), which amplify parts of the 16S rRNA encoding gene, to specifically amplify *Rhabdochlamydia* spp. The 5′-FAM probe (Eurogentec, Seraing, Belgium) contained locked nucleic acids (underlined) to improve specificity. Reactions were performed with 0.2 μM of each primer, 0.1 μM of probe, and iTaq Supermix (Bio-Rad, Rheinach, Switzerland). PCR products were detected with ABI Prism 7000 (Applied Biosystems, Rotkreuz, Switzerland). Inhibition, negative PCR mixture, and extraction controls were systematically tested.

To enable quantification, a plasmid containing the target gene was constructed as described (*4*,*9*). The analytical sensitivity of the real-time PCR for *Rhabdochlamydia* spp. was <10 copies DNA/μL. No cross-amplification was observed when the analytical specificity was tested with human, amebal (*Acanthamoeba castellanii* ATCC 30010), and bacterial DNA (Technical Appendix). Intrarun and interrun reproducibility were excellent (Technical Appendix).

This PCR and those previously described for *Parachlamydia* and *Protochlamydia* (*4*,*8*) were retrospectively applied to 39 respiratory samples from 29 neonates admitted in the neonatology unit of our institution (median 1 sample per patient, range 1–4 sample). All but 1 patient had a gestational age at birth <36 weeks (median 28.6, range 24.6–41.2 weeks). Respiratory distress syndrome was present in 25 (86%) of these 29 neonates. Samples had been drawn a median of 14 days (range 1–229 days) after birth, when clinically indicated. Results of PCR for *Parachlamydia*, *Protochlamydia*, and *Rhabdochlamydia* were positive for 9 (31%), 0 (0%), and 4 (14%) neonates, respectively. Positive results were obtained on the first sample drawn after birth for all but 2 neonates (initial negative results). One patient had positive PCR results for *Parachlamydia* and *Rhabdochlamydia*. These 12 newborns with positive PCR results for *Parachlamydia* and/or *Rhabdochlamydia* were compared with the 17 who had negative PCR results (Table).

**Table:** Characteristics of 29 newborns with positive PCR results for *Parachlamydia acanthamoebae* or *Rhabdochlamydia* spp. and controls*

Characteristics	Positive PCR result, n = 12	Negative PCR result, n = 17	p value†
Sex, M/F	8 (67)/4 (33)	6 (35)/11 (65)	0.14
Gestational age at birth, wk, median (range)	27 (24–36)	30 (25–41)	0.16
Weight <10th percentile	4 (33)	4 (24)	0.68
Height <10th percentile	3 (25)	6 (35)	0.69
Primary adaptation			
First Apgar score (1 min), median (range)	2.5 (0–7)	8 (2–9)	0.0017
First 3 Apgar scores,‡ median (range)	18.5 (8–27)	27 (17–29)	0.0023
Cardiac massage in first 48 h	6 (50)	0 (0)	0.002
Endotracheal intubation in first 48 h	11 (92)	8 (47)	0.019
Respiratory distress syndrome	11 (92)	14 (82)	0.62
Hyaline membranes disease	9 (75)	8 (47)	0.25
Bradypneic syndrome	7 (58)	11 (65)	1.00
Bronchopulmonary dysplasia	8 (67)	9 (53)	0.70
Amniotic fluid aspiration	1 (8)	3 (18)	0.62
Invasive mechanical ventilation, d, median (range)	12 (2–50)	3 (0–14)	0.005
Endotracheal intubation during hospital stay	12 (100)	11 (65)	0.028
Infectious complications			
Lung infection	5 (42)	7 (41)	1.00
Other systemic infection	7 (58)	5 (29)	0.15
Other complications			
Intraventricular hemorrhage	4 (33)	6 (35)	1.00
Persistent artery canal	7 (58)	6 (35)	0.27
Necrotizing enterocolitis	2 (17)	1 (6)	0.55
Congenital malformations	1 (8)	1 (6)	1.00
Hospitalization			
Stay in neonatology ward, d,§ median (range)	113.5 (9–435)	48 (7–131)	0.003
Death	3 (25)	0 (0)	0.06
Pregnancy			
Premature membranes rupture	5 (42)	6 (35)	1.00
Placental detachment	2 (17)	1 (6)	0.55
Preeclampsia	4 (33)	2 (12)	0.20
Systemic infection	7 (58)	9 (53)	1.00
Cesarean delivery	9 (75)	10 (59)	0.45

*Results are given as no. (%) except as indicated. †Fisher exact test and nonparametric Mann-Whitney rank sum test were used for the analysis of proportions and continuous variables, respectively. ‡Sum of the 3 scores at 1, 5, and 10 min after birth. §If survived.

Newborns with a *Chlamydia*-related organism documented in the respiratory tract had a significantly worse primary adaptation score (Apgar). These patients experienced more resuscitation maneuvers at birth. Durations of invasive mechanical ventilation and hospital stay were also longer among them. Three newborns died, compared with no deaths among the 17 with negative PCR results (p = 0.06). Pneumonia was documented in 5 of the 12 patients with positive *Parachlamydia* and/or *Rhabdochlamydia* PCR results but was concomitant to PCR positivity for only 3 of them. An alternative etiology was documented in all 3 (Technical Appendix).

*Parachlamydia* and *Rhabdochlamydia* have thus been detected in a population of premature neonates. Most of these patients had severe respiratory distress syndrome, and the role of these bacteria as a causal agent of pneumonia could not be clearly assessed. The longer duration of mechanical ventilation for newborns with positive PCR results may suggest an occult superinfection with a *Chlamydia*-related bacterium contributing to the severity of the initial respiratory disease.

Our results also raise a question about the mode of acquisition of these microorganisms. A recent study reported a higher seroprevalence of *Parachlamydia* in women experiencing miscarriage (*5*,*6*), and DNA of this bacterium has been detected in the amniotic fluid of a woman with premature delivery (*7*). Whether neonatal infection results from a systemic infection during pregnancy or an inoculation at delivery is unknown. Because of the retrospective design of the study, no samples from the mothers were available for additional molecular or serologic analyses. Hospital water supplies are an important reservoir of free-living amebae and may represent another mode of acquisition because patients undergoing mechanical ventilation are exposed to aerosolized particles (*10*). Simultaneous detection of Parachlamydia and Rhabdochlamydia in 2 patients with initial negative results and their simultaneous detection in 1 neonate supports the latter hypothesis.

In conclusion, *Parachlamydia* and *Rhabdochlamydia* DNA were detected in respiratory secretions of premature newborns with more severe conditions at birth, more mechanical ventilation requirements, and a trend toward a higher mortality rate. The pathogenic role of these *Chlamydia*-related bacteria in neonates deserves further investigations.

## Supplementary Material

Technical Appendix Bacterial species used in determining the specificity of the real-time PCR for Rhabdochlamydia spp.*
