# Supplementary material for: Parachlamydia and Rhabdochlamydia in Premature Neonates
Source: Emerg Infect Dis. 2009 Dec;15(12):2072–5. doi: 10.3201/eid1512.090267 (PMC3044521; doi:10.3201/eid1512.090267)
Supplement: Technical Appendix — Bacterial species used in determining the specificity of the real-time PCR for Rhabdochlamydia spp.* [file 09-0267_Techapp-s1.pdf]

Parachlamydia and *Rhabdochlamydia* in Premature Neonates

Technical Appendix

Table 1. Bacterial species used in determining the specificity of the real-time PCR for *Rhabdochlamydia* spp.*

Species	Source of strain
Bordetella pertussis	Clinical specimen
Chlamydia trachomatis	Clinical specimen
Chlamydophila pneumoniae	ATCC VR-1310
Criblamydia sequanensis	Strain CRIB-18
Enterococcus faecalis	ATCC 29212
Escherichia coli	ATCC 35218
Gardnerella vaginalis	Clinical specimen
Haemophilus influenzae	ATCC 49247
Klebsiella pneumoniae	ATCC 27736
Lactobacillus spp.	Clinical specimen
Legionella pneumophila	Clinical specimen
Neisseria lactamica	Clinical specimen
Neochlamydia hartmanellae	ATCC 50802
Parachlamydia acanthamoebae	Strain Hall's coccus
Parachlamydia acanthamoebae	ATCC VR-1476 (strain BN9)
Protochlamydia amoebophila	ATCC PRA-7 (strain UWE25)
Protochlamydia naegleriophila	Strain KNic
Pseudomonas aeruginosa	ATCC 27853
Simkania negevensis	ATCC VR-1471
Staphylococcus epidermidis	Clinical specimen
Streptococcus agalactiae	ATCC 13813
Streptococcus pneumoniae	Clinical specimen
Waddlia chondrophila	ATCC VR-1470

*ATCC, American Type Culture Collection.

Table 2. Clinical characteristics of 12 neonates with positive PCR results for *Parachlamydia acanthamoebae* or *Rhabdochlamydia* spp.

Patient (sex)	Gestational age at birth, wk	APGAR score*	Cardiac massage (first 48 h)	Duration of invasive ventilation, d	Species identified (d from birth)	Infections† (d from birth)	Other isolated pathogen	Hospital stay, d/ outcome
1 (F)	26	19	No	5	*Parachlamydia (*14)	Pneumonia (21)	*Enterobacter aerogenes*	90/Survived
2 (M)	35	25	No	9	*Parachlamydia* (20)	–	–	110/Survived
3 (M)	24	15	Yes	50	*Parachlamydia* (15)	Pneumonia (5)	*Ureaplasma urealyticum*	140/Survived
4 (M)	29	11	No	14	*Parachlamydia* (1)	Chorioamnionitis (5)	*Streptococcus* (gr. G), *U. urealyticum*	113/Survived
5 (M)	27	8	Yes	9 (Died)	*Parachlamydia* (7)‡	Pneumonia and chorioamnionitis (1)	*Streptococcus pneumoniae*§	9/Died
6 (F)	27	18	Yes	23	*Parachlamydia* (10)	–	–	203/Died
7 (M)	36	22	No	3	*Parachlamydia* (3)	–	–	9/Survived
8 (M)	25	11	Yes	18	*Parachlamydia* (149)	–	–	255/Survived
9 (M)	27	24	No	2	*Parachlamydia/ Rhabdochlamydia* (18)	Enterocolitis (12)	–	90/Survived
10 (M)	30	14	Yes	9	*Rhabdochlamydia* (155)‡	–	–	255/Survived
11 (F)	28	27	No	26	*Rhabdochlamydia* (229)	–	–	435/Survived
12 (F)	27	22	Yes	45	*Rhabdochlamydia* (22)	Enterocolitis (12)	–	114/Died

*Sum of the 3 Apgar scores (at 1, 5, and 10 min after birth).
†Diagnosis ≤ or ≥10 days after positive sample.
‡Previous sample with negative PCR result (for the mentioned species).
§isolated from placenta.

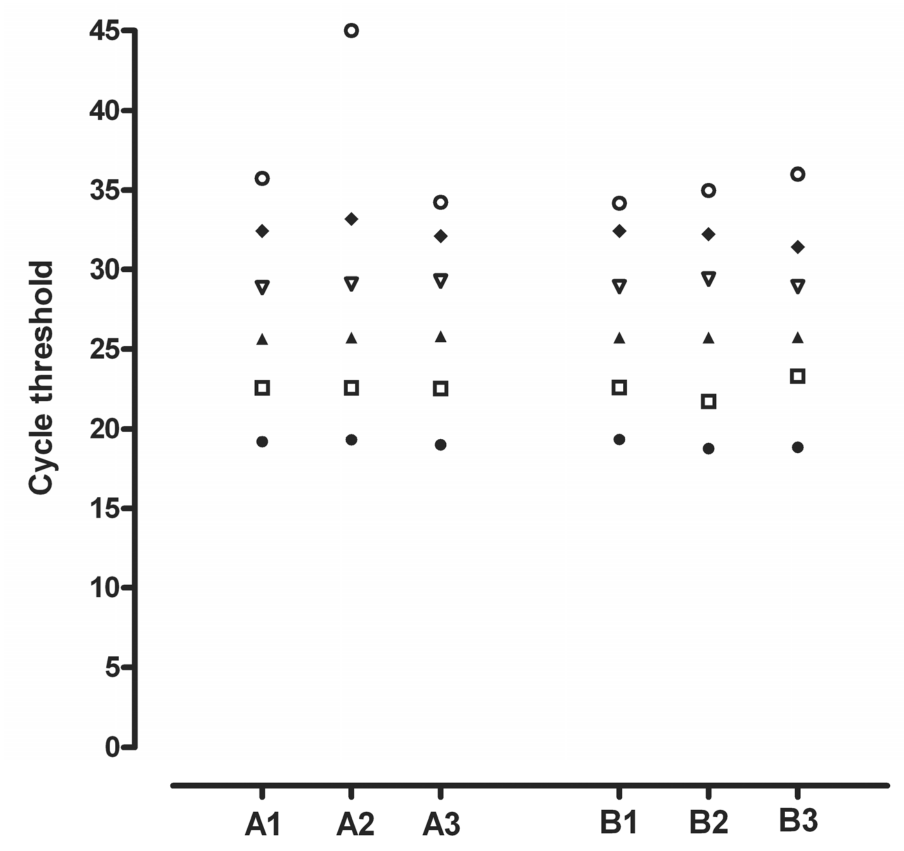

Figure. Intrarun and interrun reproductibilities of real-time PCR for *Rhabdochlamydia* spp. assessed by testing 10-fold dilutions (1–10^5 plasmid copy/μL) of plasmidic positive controls in 2 consecutive runs (A and B), each consisting of 3 duplicates. One duplicate at 10^5 plasmid/μL was negative.
